# Analysis of microstructural characteristics and components of red and yellow ink pigments used in permanent makeup

**DOI:** 10.1186/s42649-022-00072-3

**Published:** 2022-04-11

**Authors:** Hyun Sook Jin, Byung Soo Chang

**Affiliations:** Department of Cosmetology, Hanseo University, 46 Hanseo 1-ro, Haemi-myeon, Seosan, Chungnam 31962 Korea

**Keywords:** Cosmetics, EDX, Iron oxide, SEM, Semi-permanent makeup, Tattoo, Titanium dioxide

## Abstract

Our purpose in this study is to analyze the microstructural characteristics and constituent elements of inorganic substances added to the yellow ink and red ink pigments used in permanent makeup. We observed the microstructural properties of inorganic pigments added to the ink using a scanning electron microscopy (SEM) and analyzed the constituent elements of the inorganic pigment particles using an energy dispersive X-ray spectroscopy (EDX). In red wine-colored ink, cubic titanium dioxide with a diameter of 110 to 200 nm was the major component, and rod-shaped iron oxide was rarely observed. Most of the ingredients of taupe yellow ink were rod-shaped yellow iron oxide, and a small amount of cubic titanium dioxide was observed. Red wine-colored ink and taupe yellow ink contained lumps composed of titanium dioxide particles. In red wine-colored ink, lumps were formed by agglomeration. However, we observed that the surface of the lump composed of titanium dioxide in the taupe yellow ink had a smooth surface caused by external physical compression. The titanium dioxide particle mass which found in taupe yellow ink in this study is an artificial product. When this mass accumulates in the dermis, it may cause a color mismatch. Therefore, permanent makeup using fine pigments should be free of foreign substances that may cause trouble in the skin. In addition, there is a need to improve the quality of the ink so that the required color can be safe and long lasting in the dermis.

## Introduction

Tattoos have been used to show the originality of one’s appearance by decorating the skin, to mark the status of a member of a group, or as a mark of punishment. In addition, tattoos were done to improve physical beauty by camouflaging skin diseases or scars caused by surgical operations (Dorfer et al. 1999). Tattoos used for medical treatment and cosmetic purposes are named variously as micropigmentation, dermatography, permanent makeup, cosmetic tattooing, semi-permanent makeup (Jin and Chang 2019).

The public’s perception of tattoos is changing in meaning and importance with the times. Over the past few decades, the perception of tattoo procedures has changed dramatically around the world. In Europe and the Americas, body art, such as tattoos and piercings, is rapidly increasing in popularity among young people, including adolescents (Nishioka and Gyorkos 2001). In particular, in Korea, from adolescents to all age groups, there is much interest in cosmetic tattoo procedures.

Tattooing is the process of injecting exogenous pigments into the skin. In this process, pigment particles are injected into the dermal papillary layer located below the epidermis and the dermal junction, are predated by surrounding macrophages, are deposited on fibroblasts, and are permanent (Vassileva and Hristakieva 2007). Pigment particles injected into the dermis can produce a specific skin color by means of a procedure based on various skin designs.

Tattoos are also medically applied for treatment or diagnoses, as in endoscopic tattooing, corneal tattooing, and tattooed cytostatic drugs for the treatment of viral warts (Nishioka and Gyorkos 2001).

Medical tattooing can permanently camouflage a wide range of skin disorders. In addition, tattoos are used to hide scars or damaged areas as the last important step after various surgical operations, such as craniofacial surgery, plastic and reconstructive operations, cosmetic surgery, and breast reconstruction (Vassileva and Hristakieva 2007).

Cosmetic tattooing is a permanent makeup that improves the shape of eyelashes and enlarges or corrects the position of eyebrows. By means of cosmetic tattooing, the eyebrow line is formed and the eyelashes are thickened. Eyebrow tattooing gives richness to hairless eyebrows or darkens hairless eyebrow skin (Angres 1984; Patipa 1987; Oumeish 2001). In addition, cosmetic tattooing is applied to improve the contours of deformed lips after trauma or surgery or to shape the nipples.

With permanent makeup, there is no need to remove makeup for exercise or swimming. Since the tattooing is permanent, as if you had new makeup, you can save the time needed to apply makeup (De Cuyper 2008).

Permanent lip makeup is a simpler and more permanent technique than is injecting collagen into the lips to create “French” lips. For the lip liner or overall lip-color tone, micropigmentation can be used to change the size and shape of the lip and make the lip color darker. The lip lining is made step by step to achieve the effect desired by the individual. Many patients wear permanent lip tattoos to keep their lips young and clear, by means of procedures such as lip enlargement, lip lifting, and resurfacing. By these procedures, tattoos on the scarlet border of the skin and lip mucosa can be made into shiny red lips (Fulton Jr et al. 2000).

The red pigment used in permanent makeup makes the lips look thicker, adjusts the size and shape of the lips, and clearly shows the scarlet border between the lips and the mucous membrane. For permanent makeup on the lips, red dye is used alone, or bubblegum or orange pigment is appropriately added and mixed to prevent discoloration.

Yellow-based pigments are neutralizing pigments, which are mainly used to express a hue suitable for various skin colors of customers, and are used for permanent makeup for eyebrows and eyelashes.

As such, tattoos are used for various medical treatments and cosmetic purposes. However, tattoos pose a potential medical risk (Wenzel et al. 2016). The needles used in tattoo procedures may threaten the individual’s health during the skin invasion process. For tattooing of the skin, needles can be exposed to and contaminated by various blood-borne infectious diseases, which can cause serious problems in community public health (Nishioka and Gyorkos 2001).

In addition, because of the wear on the needle during tattoo procedures, metal fragments may fall off the needle and be deposited on the skin, which may lead to skin diseases, such as allergic reactions.

Micropigmentation used for permanent makeup along with needles should not be toxic to the human body, and pigments of various colors deposited in the skin after the procedure should retain their color for a long time. The injection of inorganic pigments into the skin can cause chronic and systemic diseases.

Our purpose in this study was to confirm the microstructural homogeneity of the ink pigment particles used in permanent cosmetic procedures and to identify the presence or absence of hazardous foreign substances.

We selected the red ink, which is used to make the lips look thicker and to form the outline of the lips, and the yellow ink, which is a neutral pigment used to express the color of the eyebrows and eyelash lines, as the analytes. We observed the size and shape of the pigment particles added to these materials with a scanning electron microscope, and analyzed the constituent elements by using an energy dispersive X-ray spectroscope.

## Material and method

### Experimental materials

**Fig. 1:**
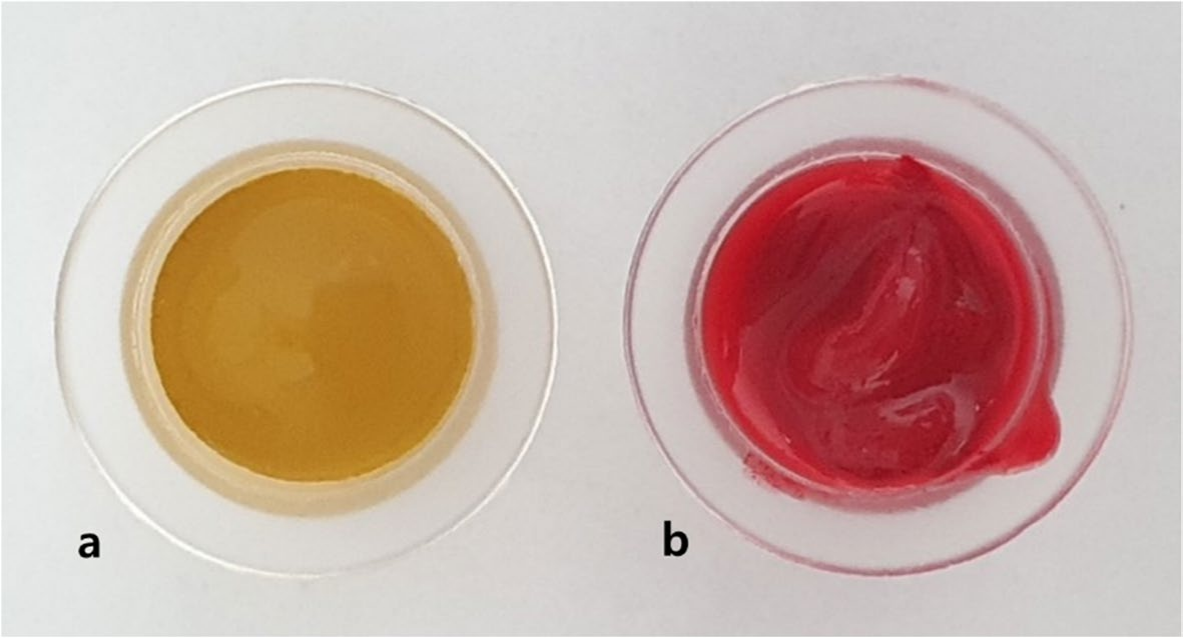
Photograph of taupe yellow colored ink (**a**) and red wine colored ink (**b**) samples used in this study

We selected one type of red ink (red wine color, MB Co., Korea), which is mainly used for lips in permanent makeup procedures, and one type of yellow ink (taupe yellow, MB Co., Korea), which is mainly used for permanent makeup on eyebrows, as experimental materials (Fig. 1).

### Method

#### Separation of inorganic substances

In order to analyze the inorganic substances added to the red wine-colored ink and the taupe yellow ink, we put 1 ml of each sample into a Falcon tube. An organic solvent (absolute ethanol, Merck, Germany) was added to this tube, diluted in a vortex mixer (MX-E, Nasco, Korea), and then precipitated. The supernatant containing organic substances was discarded, and the precipitate was stirred three times in the same manner and then precipitated. Each precipitate was used as experimental material.

#### Scanning electron microscope observation

Each sample was dropped onto a stub treated with carbon tape and copper tape and then dried naturally in order to check the types and microstructural characteristics of inorganic substances separated from the red wine-colored ink and taupe yellow ink. Subsequently, platinum was coated to a thickness of 20 nm using an ion deposition machine (IB-5 ion coater, Eiko, Japan). We then observed each sample at 15 kV with a scanning electron microscope (S-4700, Hitachi, Japan).

#### Energy dispersive X-ray spectroscopy analysis

We dropped each sample onto a stub treated with carbon tape and then dried it naturally in order to analyze the constituent elements of inorganic materials separated from the two inks. Subsequently, platinum was coated to a thickness of 20 nm using an ion deposition machine (IB-5 ion coater, Eiko, Japan). We then analyzed each sample at 15 kV acceleration voltage with energy dispersive X-ray spectroscopy (INCA, Oxford, Inc., Great Britain).

## Result

**Fig. 2:**
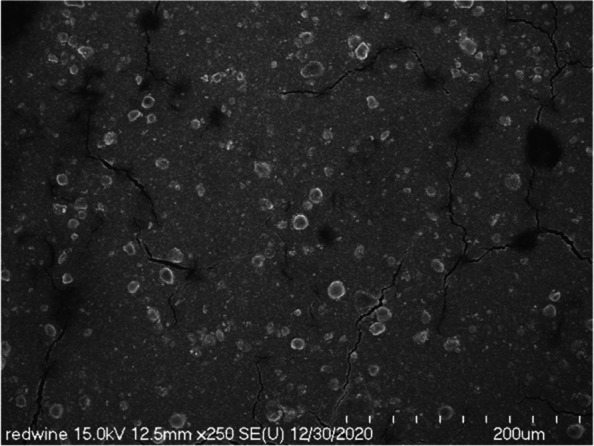
Low magnification scanning electron micrograph of red wine-colored ink pigments. Note that lumps of various sizes and shapes are formed

**Fig. 3:**
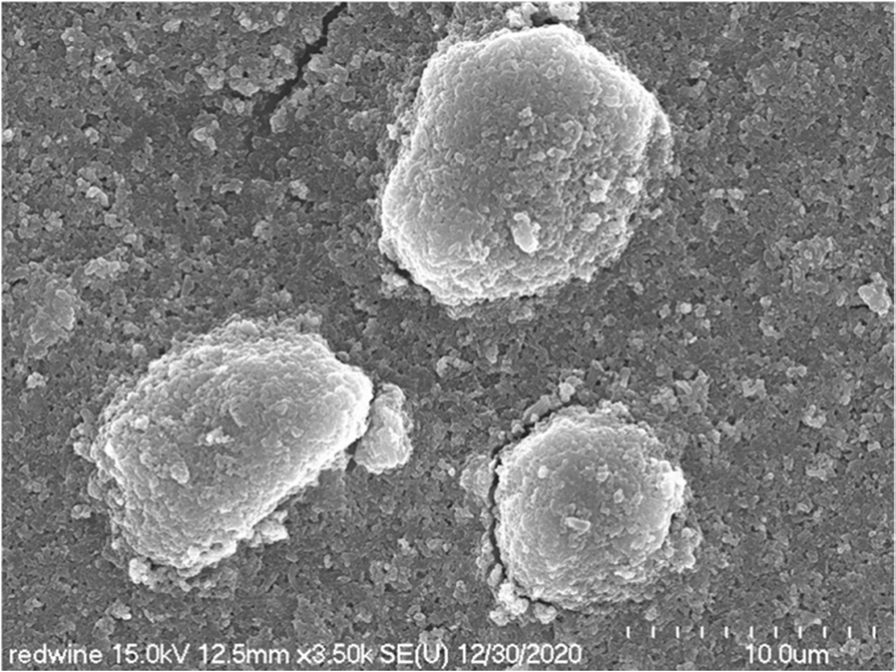
Scanning electron micrograph of red wine-colored ink pigments showing spherical and elliptical lumpy matters

We observed the red wine-colored ink, which is mainly used for lip tattoos, with a scanning electron microscope at a low magnification of 250 times. We found out whether the particles were evenly dispersed or were gathered to form a lump (Fig. 2). On the scanning electron microscope at 3500 times magnification, the types or characteristics of evenly dispersed particles and particles forming agglomerates were not identified (Fig. 3). Most of the lumps were spherical or elliptical, and the size, measured in various ways, was up to 12 μm in diameter.

**Fig. 4:**
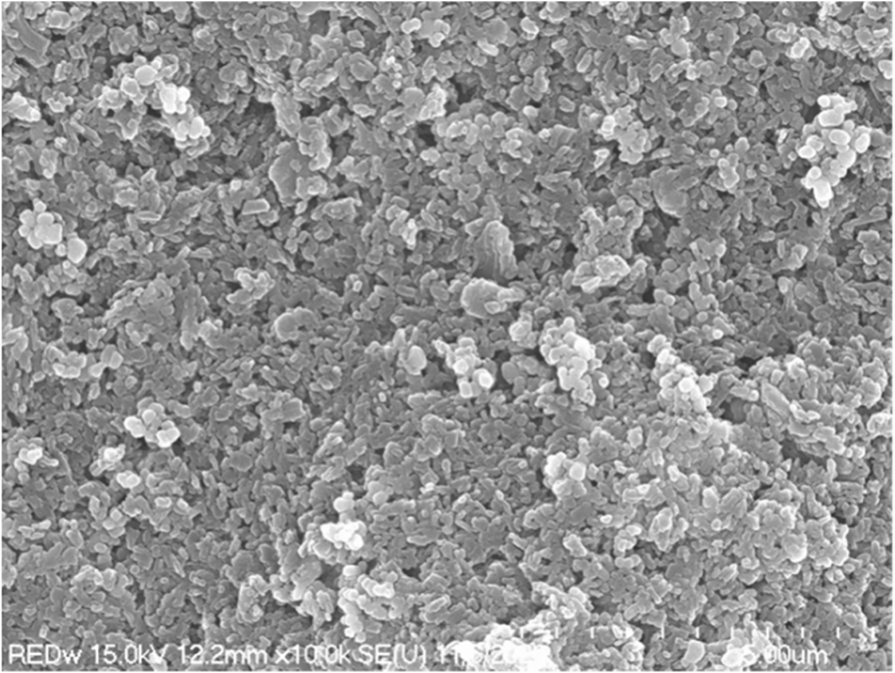
Scanning electron micrograph showing cuboidal and spherical shaped pigments red wine-colored ink

**Fig. 5:**
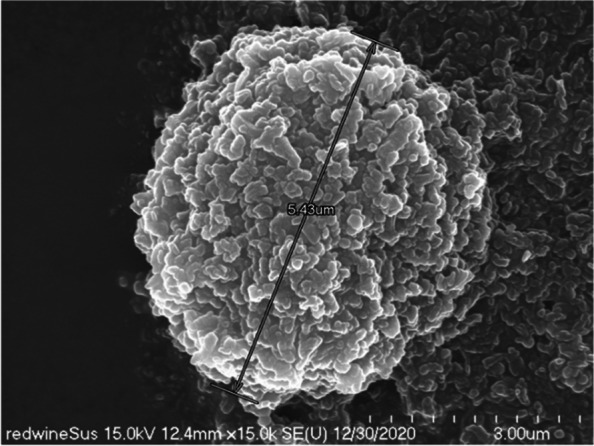
Scanning electron micrograph of red wine-colored ink pigments showing aggregated cuboidal and spherical particles. Clumped materials have a diameter of about 5.4 μm

On a scanning electron microscope at 10,000 times magnification, we observed that the fine pigment particles were evenly dispersed (Fig. 4) and confirmed that the lump was composed of the same pigment particles. It was about 5.4 μm in diameter (Fig. 5).

**Fig. 6:**
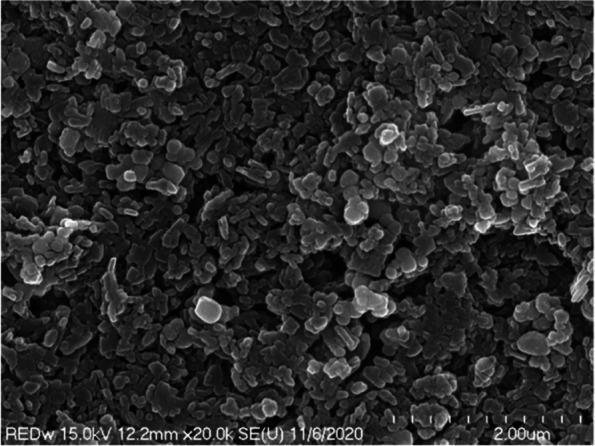
Scanning electron micrograph of cuboidal and spherical particles added to red wine-colored ink

**Fig. 7:**
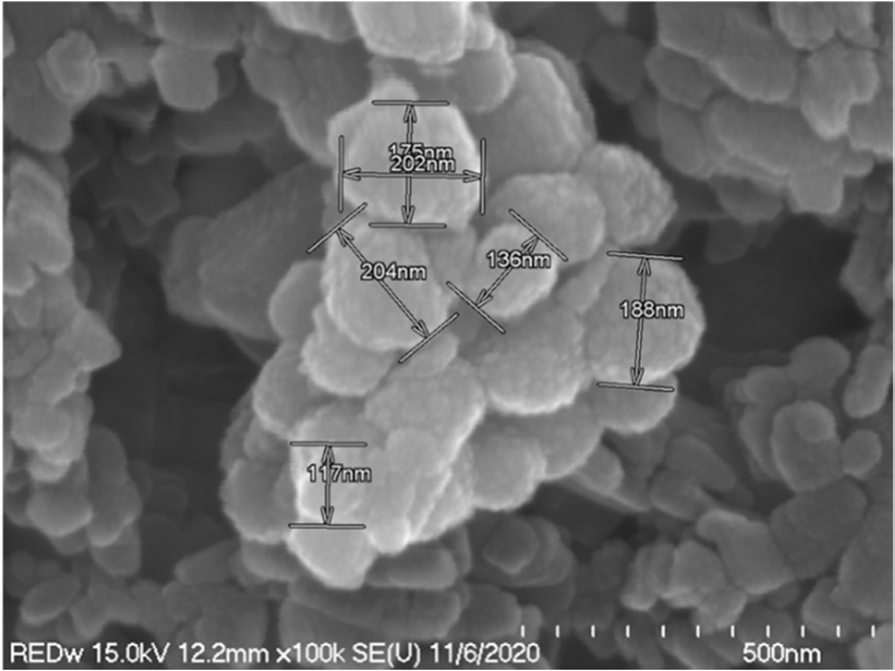
High-magnification scanning electron micrograph of red wine-colored ink particles showing aggregated titanium dioxide. The titanium dioxide size shows a cubic shape with a diameter of about 110–200 nm

In high-magnification scanning electron microscopy, we identified the dispersed particles as spherical, cubic, or rod-shaped (Fig. 6). We identified the cubic particles as titanium dioxide, and the rod-shaped particles as iron oxide. Titanium dioxide was agglomerated in a small amount in a dispersed state, or a large number of particles were agglomerated to form a lump. As seen on the high-magnification scanning electron microscope, titanium dioxide was cubic and had a diameter of about 110 to 200 nm (Fig. 7).

**Fig. 8:**
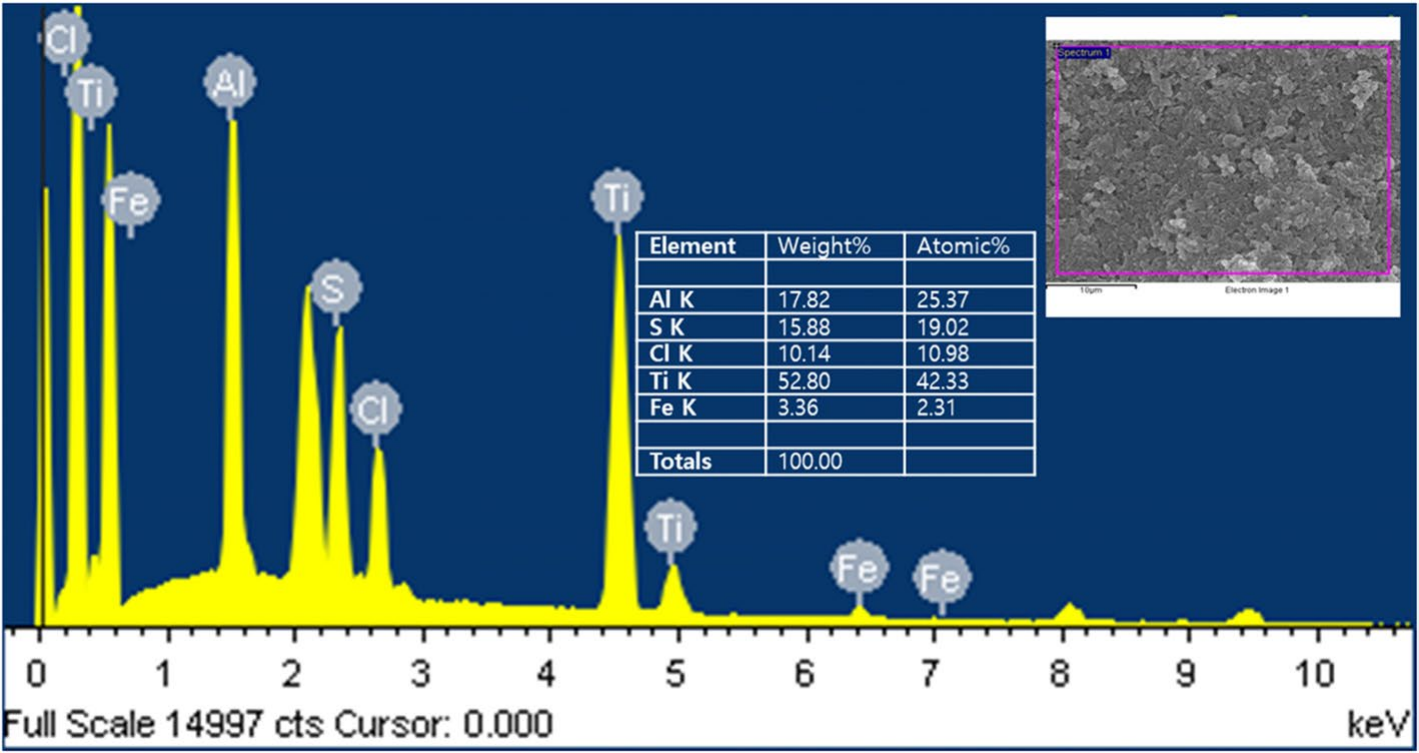
EDX spectrum of red wine-colored ink pigments sample. EDX reveals Ti, Al, S, Fe, Cl. Inset: Content of the constituent elements of red wine-colored ink pigment

We did EDX analysis to assay constituent elements of inorganic substances added to red wine-colored ink. In this experiment, we detected elemental components of titanium (Ti), aluminum (Al), sulfur (S), chlorine (Cl), and iron (Fe). The components detected in the red wine-colored ink pigment were titanium (Ti) 42.33%, aluminum (Al) 25.37%, sulfur (S) 19.2%, and iron (Fe) 2.31% (Fig. 8). Thus, scanning electron microscopy and EDX analysis identified that the pigment particles of red wine-colored ink were mostly cubic or spherical titanium dioxide and found rod-shaped iron oxide to be present in a small amount.

We observed taupe yellow ink pigment, used as a neutralizing pigment to correct skin color during eyebrow and eyelash tattoo procedures, with a scanning electron microscope.

**Fig. 9:**
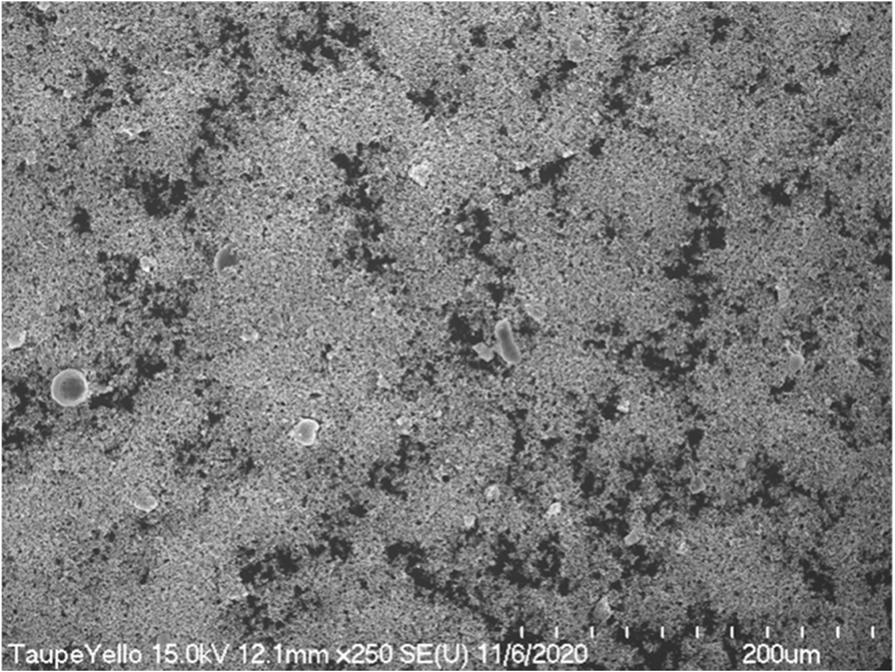
Low-magnification scanning electron micrograph of taupe yellow ink pigments

**Fig. 10:**
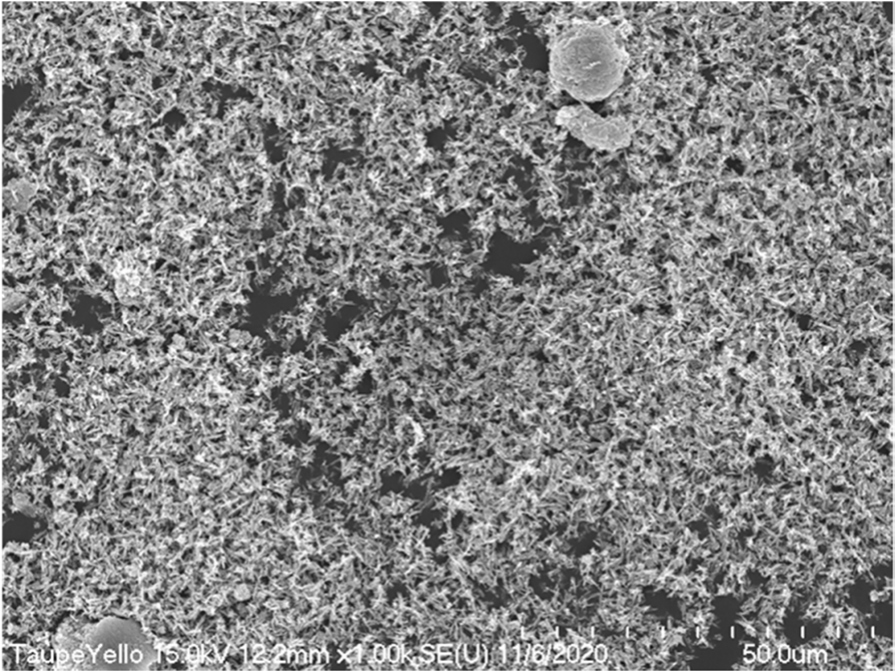
Scanning electron micrograph of taupe yellow ink pigments showing rod-shaped iron oxide and lumped materials

In scanning electron microscopy observation at a low magnification of 250 times, we observed the taupe yellow ink pigment in the form of some lumps in a state where fine particles were dispersed (Fig. 9). The lump was spherical or rectangular but also had some irregular shapes. At 1000 times magnification scanning electron microscopy, we observed the pigments to be evenly dispersed; most of them were identified as rod-shaped iron oxides (Fig. 10). In addition, we observed spherical or irregularly shaped lumps. However, the pigments constituting the lump were not identified at low magnification.

**Fig. 11:**
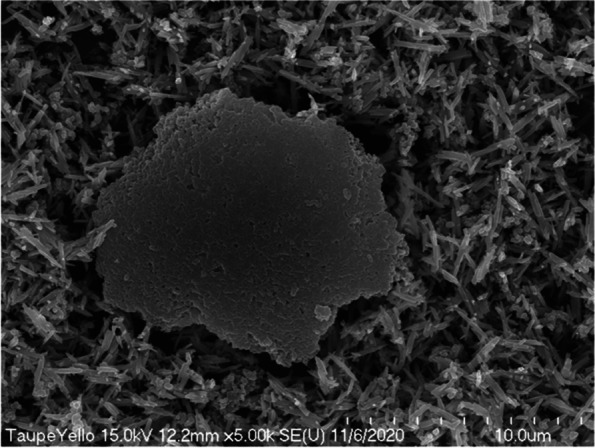
Scanning electron micrograph showing rod-shaped iron oxide and lumped titanium dioxides added to taupe yellow inks

**Fig. 12:**
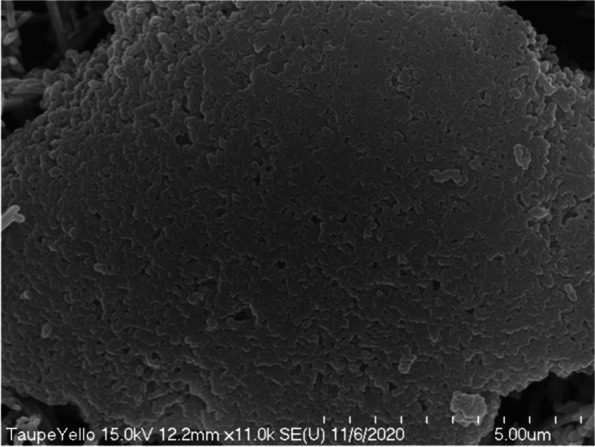
Magnified scanning electron micrograph of Fig. 10 showing titanium dioxides aggregated lumpy material

The lump-shaped structure was physically compressed and was about 15 μm in diameter (Fig. 11). Around this lump, there were rod-shaped iron oxides that were evenly dispersed. In the magnified image of the lump in Fig. 10, we observed that the surface of the lump that appeared to be formed by physical compression was composed of pigments having the same shape (Fig. 12). These particles had the properties typical of titanium dioxide.

**Fig. 13:**
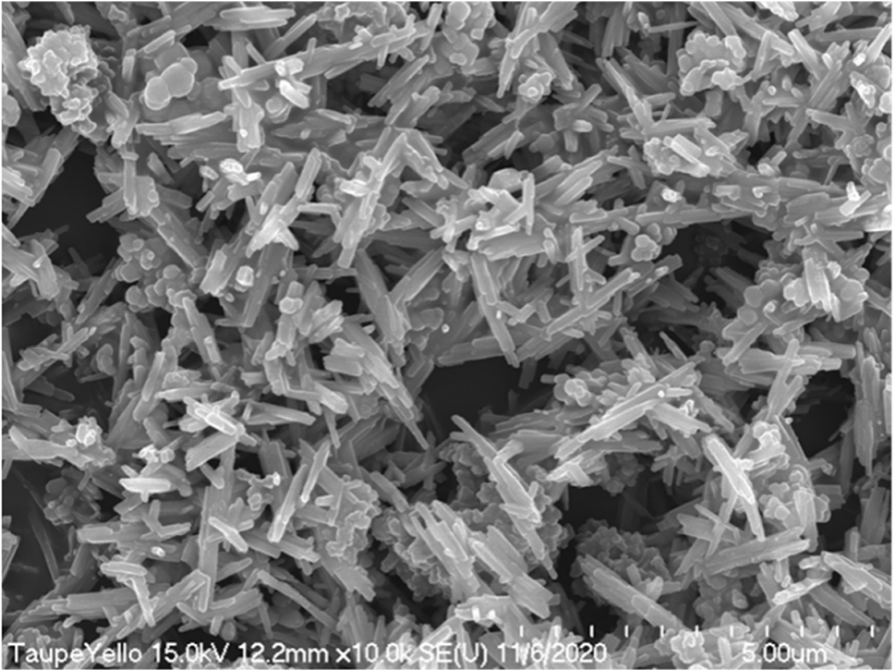
Scanning electron micrograph showing rod-shaped iron oxide and cuboid titanium dioxide added to taupe yellow inks

**Fig. 14:**
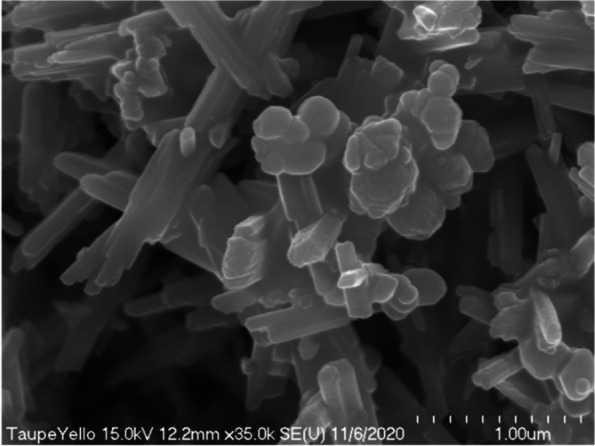
High-magnification scanning electron micrograph showing rod-shaped iron oxide and cuboid titanium dioxide added to taupe yellow inks

In scanning electron microscope observation, we observed many rod-shaped iron oxides and confirmed that a small amount of cubic titanium dioxide was clustered and scattered between iron oxides (Fig. 13). In the high-magnification scanning electron microscope observation, the rod-shaped iron oxide was about 670 nm long; and the titanium dioxide particles were not separated from each other, but a few titanium dioxide particles were aggregated (Fig. 14).

**Fig. 15:**
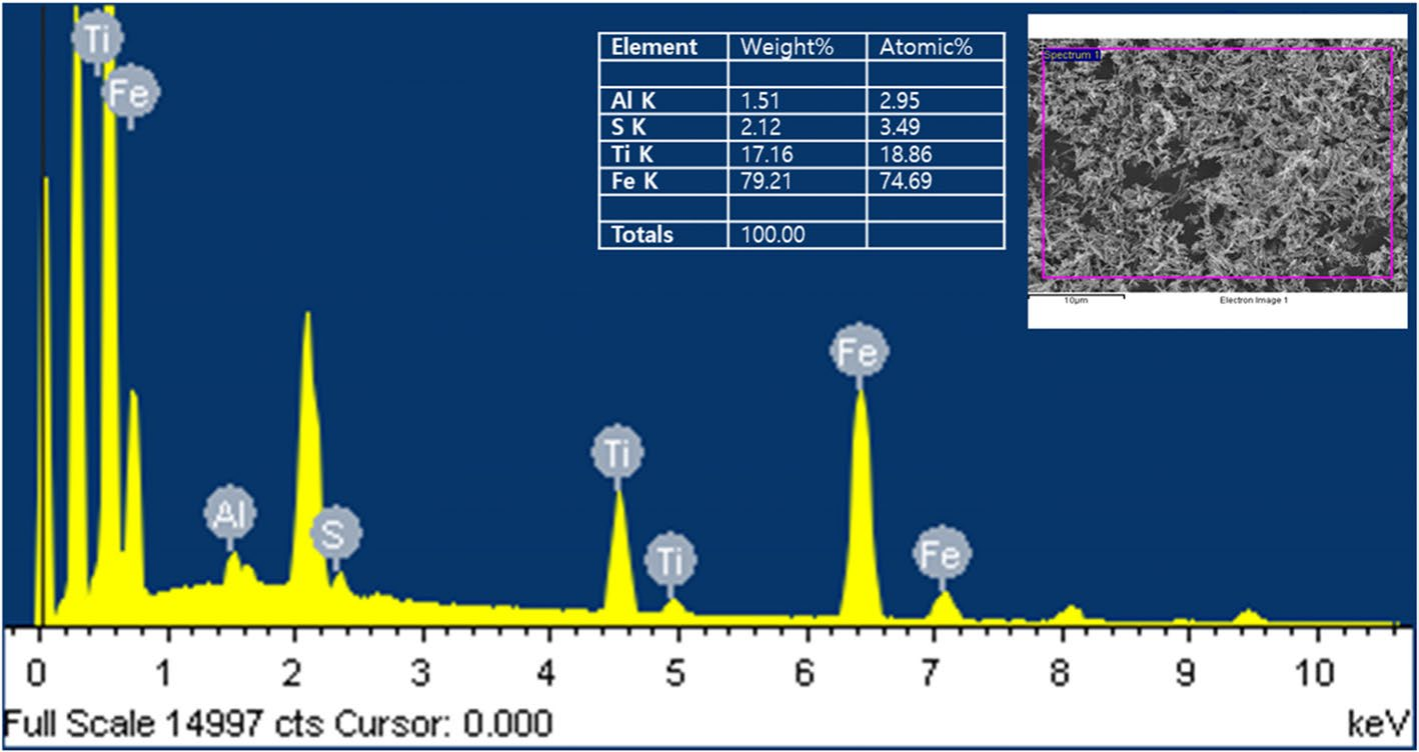
EDX spectrum of taupe yellow ink pigments sample. EDX reveals Fe, Ti, S, and Al. Inset: Content of the constituent elements of taupe yellow ink pigments

To analyze the constituent elements of the taupe yellow ink pigment, we used EDX analysis and detected the following elemental constituents: Iron (Fe), titanium (Ti), sulfur (S), and aluminum (Al). The elements added to the taupe yellow ink pigment were 74.69% iron (Fe), 18.86% titanium (Ti), 3.49% sulfur (S), and 2.95% aluminum (Al). EDX and observation with a scanning electron microscope confirmed that the taupe yellow ink pigment was mainly composed of rod-shaped iron oxide, followed by cubic titanium dioxide (Fig. 15).

## Discussion

Recently, permanent makeup has been in the spotlight as a beauty art for the human body that expresses beautiful faces by forming eyebrow lines, strengthening eyelashes, and permanently changing the shape of lips (Montgomery and Parks 2001; Wetzel 2012; AlQuorain et al. 2017). The areas where permanent makeup is most commonly applied as cosmetic tattooing are eyebrows and eyelashes, injecting pigments into the lip line and the entire lip, rouge of cheeks, and beauty spots (Wetzel 2012).

The yellow ink, which is commonly used in eyebrow and eyelash treatment, acts as a neutralizing pigment that corrects the ink material so that it is close to the customer’s skin color, prevents discoloration of the ink material after the treatment, and extends the maintenance period. The red pigment plays a role in making the lips look thicker and sharpening the lip line, making the lips attractive.

In this study, by observing the red wine-colored ink pigment of the red families with a scanning electron microscope, we found that the pigment particles were evenly dispersed or gathered to form a lump. We identified the cubic particles as titanium dioxide, with a diameter of about 110 to 200 nm. The rod shapes were iron oxide and were about 600 to 700 nm long.

Most of the lumps were spherical or elliptical. The titanium dioxide was lumped together into lumps of a variety of sizes, from about 5.4 to 12 μm.

Titanium dioxide’s agglomeration is why the particles can easily accumulate on the skin during permanent makeup procedures. The desired color tone can be obtained only when the ink solution on the tip of the sharp needle accumulates without being dispersed or lost on the application area of the skin. Titanium dioxide provides the most commonly used nanoparticles for industrial paints, paper, textiles, plastics, and cosmetics. It is a white pigment with low toxicity and has anti-corrosion characteristics and high photocatalytic activity (Kotil et al. 2017).

Yellow ink is used as a neutralizing pigment to harmonize with the client’s skin color when performing permanent makeup on eyebrows and eyelashes.

Our observations in this study confirmed that rod-shaped iron oxide and cubic titanium dioxide were dispersed in a taupe yellow ink pigment, and that there were lumps of titanium dioxide between the iron oxide and titanium dioxide particles. Iron oxide particles were about 670 nm long and had a uniform shape.

Iron oxide is classified into rod-shaped iron oxide red, iron oxide yellow, and cubic iron oxide black. Red iron oxide and yellow iron oxide are similar in size and shape, so they are not easily distinguished by scanning electron microscopy (Jeon and Chang 2010).

In this study, yellow iron oxide added to taupe yellow ink had the same shape and size. Iron oxide is a fine powder with cohesive force, forms a large surface area, and has a magnetic field; so it has a high surface energy (Zheng and Zhang 2003).

In this study, iron oxide exhibited agglomeration, which helps facilitate the deposition of pigment particles on the skin and minimize loss of particles during permanent cosmetic procedures.

In particular, we observed many lumps formed by the physical compression of titanium dioxide particles in the taupe yellow ink pigment. These agglomerated surfaces were very smooth. We assumed that such a compacted surface lump is an artifact produced when the titanium dioxide raw material is manufactured.

As described above, when a compressed lump of 15 μm in the ink pigment is accumulated in the dermal surface layer, it becomes difficult to express skin tone, and skin disease may also be caused. The pigment component added to the ink is not soluble in water or organic solvents, and the sizes of the particles are very irregular. Other substances harmful to the human body should not be added.

Beauty tattoos were invented before the modern era and are not a trend that temporarily becomes fashionable and then disappears. Tattooing must be done by a professionally trained practitioner using advanced medically certified equipment and needle cartridges with skilled techniques (Goldstein 2007).

In particular, non-toxic inks and inks that can last a long time in various colors must be developed continuously. Ultimately, tattoos should be medically safe and have no side effects after the procedure. Tattoos should help the person get an artistically pleasing image and help the person who wears the tattoo live a beautiful life.

The elements detected in inorganic substances added to red wine-colored ink were titanium (Ti), aluminum (Al), sulfur (S), chlorine (Cl), and iron (Fe). The elements detected in the taupe yellow ink pigment were iron (Fe), titanium (Ti), sulfur (S), and aluminum (Al). Iron (Fe). Titanium (Ti), sulfur (S), and aluminum (Al) were commonly present in the two ink pigments used in this study.

Semi-permanent cosmetic ink is composed of insoluble pigments dispersed in water and additives, such as dispersants and preservatives, and contain some flavorings. The most important component in semi-permanent cosmetic ink is a colorant, which is an insoluble pigment. Most tattoo inks consist of carbon black, colorants such as titanium dioxide and iron oxide, and auxiliary substances (Laux et al. 2016).

The color development of certain shades is affected by the number of metals added to the ink and by the purity of these metals (Bocca et al. 2018). Our analysis by scanning electron microscope observation and energy dispersive X-ray spectroscope confirmed that the main pigment particles in red wine-colored ink were titanium dioxide, and iron oxide was the main elemental component in taupe yellow ink pigment.

## Conclusion

In this study, we compared and analyzed the microstructural characteristics and constituent elements of red wine-colored ink and taupe yellow ink used in permanent makeup. Iron oxide and titanium dioxide were present in each ink. Red wine-colored ink contained the most titanium dioxide, and taupe yellow ink contained iron oxide as the main component. In the red wine-colored ink, titanium dioxide was agglomerated to form lumps, with a diameter of 5 to 12 μm. We observed titanium dioxide lumps in taupe yellow ink as a structure formed by physical compression with a very smooth surface. The titanium dioxide lumps found in the taupe yellow ink had a smooth surface and were about 15 μm in diameter. Iron (Fe), titanium (Ti), aluminum (Al), and sulfur (S) were commonly detected in the analysis of constituent elements of taupe yellow ink and red wine ink pigments. However, trace amounts of chlorine (Cl) were detected in red wine-colored ink.

## Data Availability

No applicable.
